# Diversity, Equality, and Inclusion in the naïve T Cell Receptor Repertoire

**DOI:** 10.1111/imr.70138

**Published:** 2026-07-01

**Authors:** Isabella Sodi, Matthew V. Cowley, Trupti Gore, James Henderson, Andreas Tiffeau‐Mayer, Benny Chain

**Affiliations:** ^1^ Institute of Infection, Immunity, and Transplantation UCL London UK; ^2^ Department of Computer Science UCL London UK

## Abstract

The naïve T cell receptor (TCR) repertoire forms the immunological background from which adaptive cellular immune responses emerge. We examine the fundamental properties of the human naïve TCR repertoire through the perspective of Diversity, Equality, and Inclusion. We first consider the richness of the repertoire. A combination of experimental and computational approaches has been used to estimate that the human repertoire contains at least 100 million distinct naïve clonotypes. This estimate suggests an average size of each clonotype of 1000 T cells. However, evidence from both mathematical modeling and large‐scale single‐cell sequencing indicates that clonotype family sizes are very unequal, with a small subset of naïve TCRs present at substantially higher frequencies. Somatic recombination itself does not contribute significantly to this inequality, as the probability of generating an identical clonotype multiple times in an individual is very small. Instead, clonotype size is likely to be largely driven by thymic or post‐thymic expansion, but the mechanisms driving heterogeneity remain poorly understood. Finally, despite clonal deletion being a cornerstone of immunological dogma, experimental evidence for functional “holes” in the naïve repertoire caused by negative thymic selection is surprisingly limited. Alternative tolerance mechanisms, including regulatory T cells and T cell quorum sensing, are likely to play important roles. This review highlights the need for further research to identify the mechanisms that shape the frequency distribution of naïve TCR clone sizes and to define its impact on primary immune responses. Further research is also needed to understand the role of quorum sensing in maintaining T cell tolerance, while avoiding potential vulnerabilities arising from extensive ‘holes’ in the TCR repertoire.

## 1 Introduction

**FIGURE 1:**
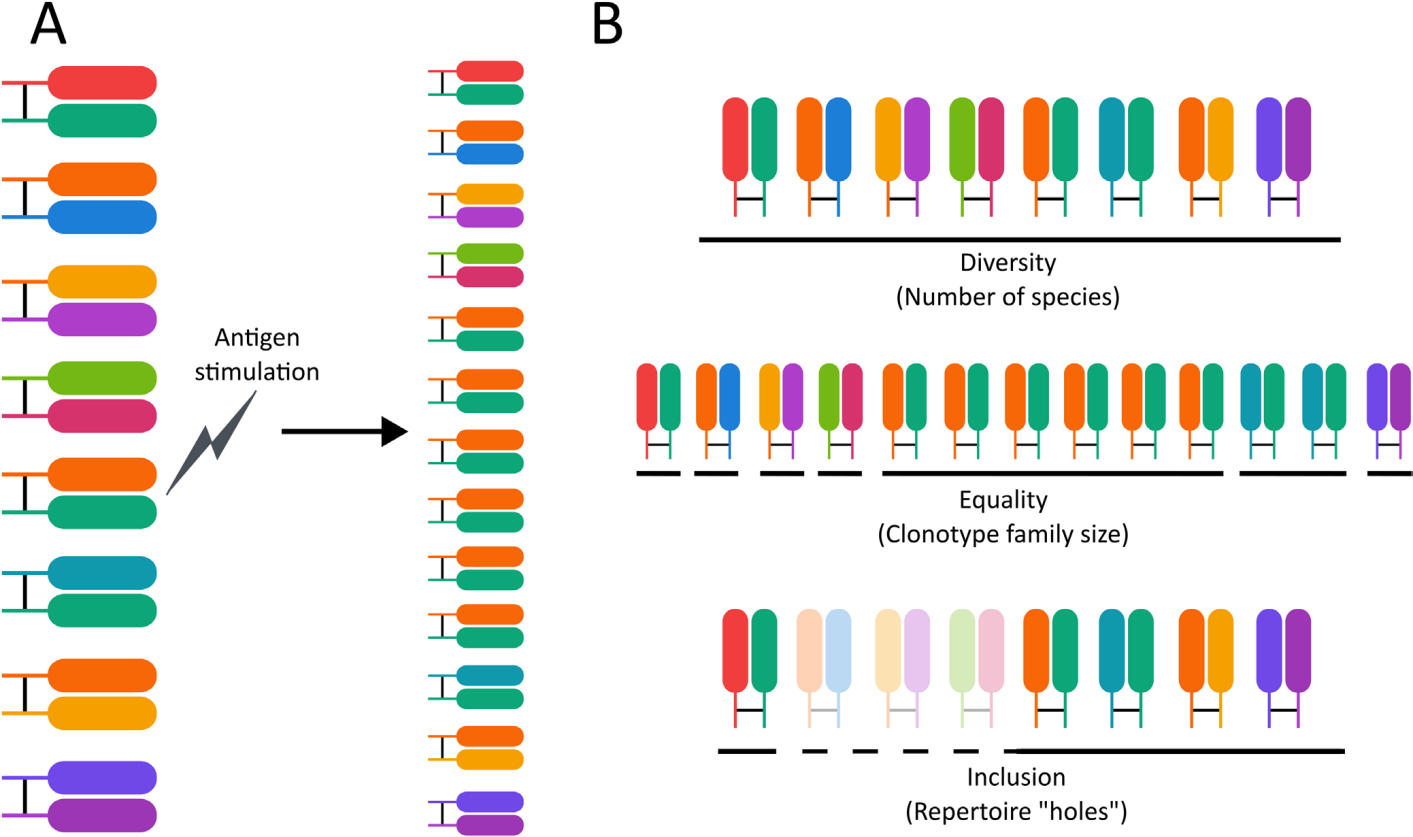
(A) Clonal expansion. The naïve repertoire consists of T cells, each of which carries a different receptor. Each T cell is present rarely. After antigen exposure, the T cells carrying cognate receptors for that antigen are expanded as a proportion of the total (increased clonal frequency). (B) Three parameters that shape and limit the naïve TCR repertoire. The somatic recombination, which generates TCR **diversity**, creates a large number of distinct TCRs that make up the repertoire (richness). **Equality** refers to the frequency distribution of naïve TCRs—how many copies of T cells, each with a unique TCR, are present within the repertoire. **Inclusion** examines how many TCRs are generated, but then excluded, creating ‘holes’ in the repertoire, due to self‐tolerance or aging.

The current model of adaptive immunity rests on the concept of clonal expansion. The key idea is that the adaptive immune system is made up of numerous lymphocytes, each of which expresses a set of identical antigen receptors (a clonotype). The antigen receptor has a pre‐determined specificity, which depends on the structure of its antigen‐binding surface. Before meeting an antigen, lymphocytes are considered antigen ‘naïve’, and each receptor is only present very rarely in the population. After exposure, the individual cells whose receptors recognize and bind the target start proliferating in response to the antigen stimulation, giving rise to ‘memory’ or ‘effector’ populations (Figure 1A). Because of the proliferation, the number of lymphocytes specific for that antigen increases as a proportion of the total population. This process is termed clonal expansion and results in increased clonal frequencies in memory and effector populations. This framework was originally developed in the context of antibody production and B cells [1], but with the discovery of the T cell receptor (TCR) [2, 3], the concept was easily extended to cellular immunity.

The receptor repertoire of the naïve T cell provides the ‘blank canvas’ for cellular adaptive immunity, which will constrain and focus the range of protective or pathological immunological responses that an individual can mount during their lifetime. In this review, we survey key processes that limit and shape the naïve TCR repertoire, focusing predominantly on human data, although some work on the mouse has also been included where appropriate. We first introduce the distinction between a functional clonotype and a molecular clonotype, and examine experimental estimates of the total number of both of these in the human repertoire. We then examine three biological mechanisms (Figure 1B) which shape and limit the repertoire: Generation of ***Diversity*** by somatic recombination, clonal expansion driving unequal expansion of naïve clonotypes (***Equality***), and negative selection creating gaps or ‘holes’ in the naïve repertoire (***Inclusion***). As we will show, despite decades of work and ever more powerful DNA sequencing technologies, considerable uncertainty still surrounds many aspects of these fundamental processes of T cell biology.

Before we start the discussion, we introduce a technical but important point. The information on T cell receptor repertoires almost always comes from nucleic acid (DNA or RNA) sequencing. Individual TCRs can therefore be identified by their nucleic acid sequence. Alternatively, the nucleic acid sequence can be translated into the corresponding amino acid sequence. Because of the redundancy of the genetic code, these two properties are not the same, and in general, many different nucleic acid sequences will give rise to the same protein sequence. The function of the T cell receptor and its specificity are determined purely by the amino acid protein sequence, and differences in the underlying DNA sequence, which do not change the protein sequence, are termed synonymous and usually considered functionally silent. For this reason, unless specifically stated, the discussion in this chapter focuses predominantly on predicted protein rather than nucleic acid sequence, even though almost all raw data available is nucleic acid‐based.

### 1.1 The Richness of the naïve Repertoire

**FIGURE 2:**
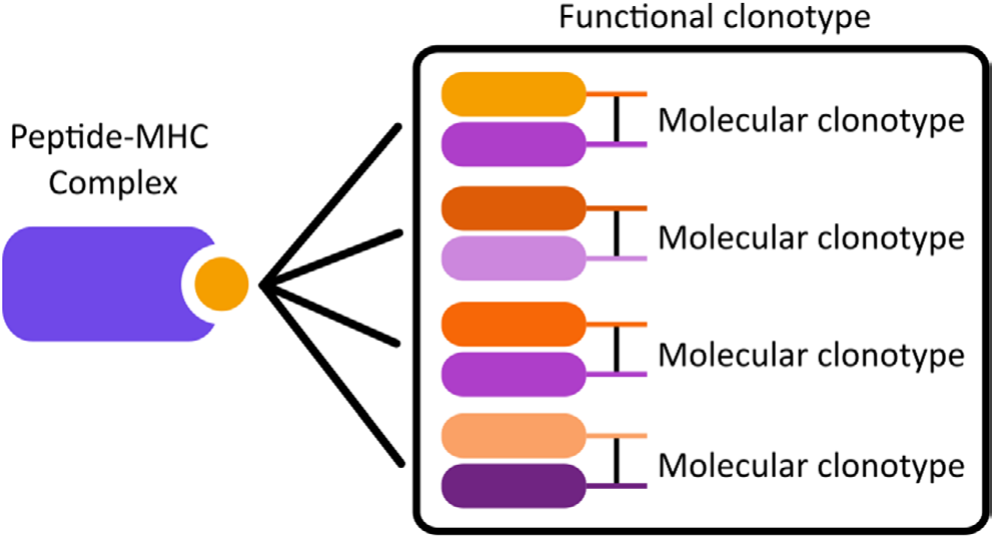
A schematic representation comparing molecular and functional clonotypes. A molecular clonotype is a unique TCR alpha/beta sequence specific for a given peptide/MHC complex. A functional clonotype is a set of molecular clonotypes in an individual that all share specificity for a given peptide/MHC.

We begin with a consideration of the number of unique TCR protein sequences realized in any given individual repertoire, a metric known in an ecological context as repertoire richness. Prior to the advent of high‐throughput T cell receptor sequencing, a number of studies estimated the size of a ‘functional clonotype’, namely the frequency of T cells specific for individual epitopes (peptide/MHC complexes) in the naïve T cell repertoire (Figure 2). It is important to note that such functional clonotypes are likely to be made up of many different clonotypes defined at protein sequence level (molecular clonotypes), a distinction discussed in more detail below. In mice, a direct approach of physically isolating the majority of the T cells in the animal is possible (reviewed in [4, 5]). In humans, the measurements are necessarily done on a small sample, typically a few million T cells from a blood draw. The observed frequencies of the T cell populations varied by several orders of magnitude between epitopes, generally ranging between 0.1–500 in 10^6^. If each set of TCRs were specific to a single epitope, this would suggest a maximum functional size of around 10^5^–10^6^ target epitopes. The observation that individual TCRs can recognize several distinct epitopes [6, 7] increases this number. Interestingly, the range of frequencies observed was broadly similar in human and mouse, suggesting this degree of diversity may correspond to some evolutionary optimum in achieving maximum protection against infection, while minimizing metabolic and genetic cost. Since the number of naïve cells in a human is some 10^4^ larger than the number in a mouse, while the functional clonotype frequency is similar, the average number of naïve T cells making up each functional clonotype must also be larger by a factor of 10^4^. The number of T cells per functional clonotype, therefore, scales approximately with weight, thus ensuring a similar basal concentration of receptors in both species.

An important caveat to these experiments lies in unambiguously distinguishing between naïve and memory populations, since effective clonotype sizes in memory populations are orders of magnitude larger than in the naïve repertoire. One approach is to rely on the known history of antigen exposure and assume that if an animal or human has not previously encountered a specific antigen, the T cell repertoire for this antigen will be naïve. For humans, it is usually impossible to know the full exposure history of an individual. In addition, the possibility of unknown cross‐reactions between antigens is a serious confounder [8, 9]. One approach to this potential confounder is to sequence the receptor repertoire of T cells from umbilical cord blood [10] which is composed primarily of naïve T cells and is presumably subject to very limited foreign antigen exposure. Paradoxically, this approach found higher frequencies of antigen‐specific T cells, perhaps reflecting a limited development of the neonatal repertoire or more promiscuous recognition by close‐to‐germline clonotypes formed preferentially in early T cell development [11]. An alternative is to rely on known phenotypic markers, often cell surface markers, to physically separate naïve and memory cells [12, 13]. The identification of the selective expression pattern of CD45 isoforms between different T cell subsets [14] combined with cell surface markers including the adhesion receptor CD62L and the chemokine receptor CCR7, has provided a robust phenotype for identifying naïve cells for many years [15]. However, it subsequently became clear that this model was oversimplified. For example, the naïve CD45RA/CD62L high population was found to include a population of ‘memory stem’ cells, T cells which had responded to external antigen, but which retained a naïve phenotype [16]. Although CD95 showed utility for distinguishing between truly naïve and stem memory phenotypes, it is difficult to exclude the presence of further small antigen‐experienced sub‐populations with a ‘naïve’ phenotype [17]. Single‐cell transcriptomics, which gives an even more detailed and high‐dimensional phenotype, allows delineation of additional heterogeneity within the naïve compartment [18].

The size of a functional clonotype, i.e., the number of cells that recognize a specific pMHC complex, does not determine the size of individual molecular clonotypes, the number of naïve T cells with a specific protein sequence (Figure 2). Many of the studies that have rigorously examined the size of functional clonotypes reactive to diverse epitopes were carried out before the era of efficient single‐cell sequencing and therefore do not report accurate estimates of the number of distinct clonotypes within the set of cells which bind each antigen. Instead, a number of studies have attempted to estimate the total number of TCR clonotypes by sequencing individual alpha or, more usually, beta chains from a sample and extrapolating. Early estimates suggested a total number of approximately 2 × 10^6^ distinct clonotypes in mice [19], each present on average 5–10 times, and 2.5 × 10^7^ distinct clonotypes in humans, each present on average 10^3^–10^4^ times [20]. Considering these numbers in relation to the functional clonotypes discussed above would suggest that each functional clonotype was made up of only 1 or 2 molecular clonotypes in mice, and 10–50 molecular clonotypes in humans. Arstila et al. [20] derived the estimate in humans by measuring the richness on the single beta‐chain level, inferring that each beta‐chain pairs with roughly 25 alpha chains and extrapolating from there. Later studies used higher throughput sequencing technologies still restricted to the beta‐chain and applying the same alpha chain extrapolation as previously [20]. The increased depth of sequencing led to 2–100 fold higher estimates for the number of distinct clonotypes in humans [21, 22]. The higher molecular clonotype estimate would correspond to more diverse clonotype repertoires for functional clonotypes recognizing individual peptide antigens.

**FIGURE 3:**
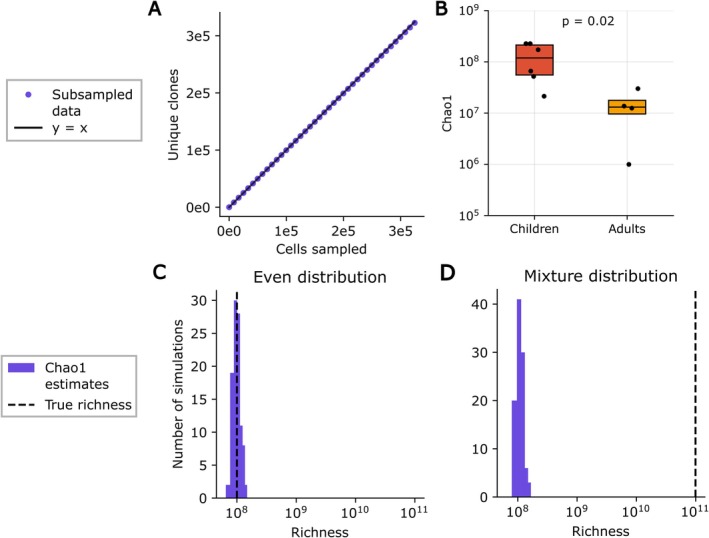
Estimates of richness from observed naïve repertoires in the TABLO dataset [18]. (A) Random subsampling of naïve cells from a representative donor demonstrates a linear relationship between the number of cells sampled and the number of unique clones identified. (B) The Chao1 index for naïve cells is larger for children than for adults. Donors above the age of 10 are considered adults. Cells that were originally considered ‘naïve’ but showed a different gene expression from truly naïve cells were not included. (C) Simulation of a uniform repertoire of 10^8^ clones with only one cell per clone. Poisson sampling at a depth of 10^5^ cells shows that the Chao1 estimate of richness works well for a uniform distribution. (D) Simulation of an uneven repertoire of 10^11^ cells, where 99% of the repertoire is singletons and 1% is expanded to 10^5^ cells per clone. Poisson sampling at a depth of 10^5^ cells shows that the Chao1 estimator is a lower bound on the true richness.

The studies above sequenced TCR alpha and beta chains individually, and assumptions were made about how many alpha chains paired with a single beta to arrive at an estimate of molecular clonotype richness. Single‐cell sequencing of TCR repertoires can overcome the limitations of bulk sequencing approaches by resolving alpha‐beta pairing and hence identifying true clonotypes. However, typical studies use samples too small to infer any meaningful information about naïve repertoire richness. Recently, Sureshchandra et al. deeply profiled both the global transcriptome and T cell receptors of 5.7 million T cells from the blood and tonsils of 10 donors [18]. This data resource, called TABLO, provides a T cell atlas derived from blood and tonsil cells, in which naïve cells can be identified based on their gene expression profiles. To derive naïve richness estimates, we used the naïve phenotype assignments from the original study, while excluding a cluster of cells with higher rates of clonally expanded cells (as discussed in detail in the next section). Rarefaction analysis of the naïve repertoire shows that with (nearly) every cell sampled, an additional clone is detected, consistent with prior estimates of high clonal richness (Figure 3A). However, there is a small (but detectable) minority of clones, where multiple cells are sequenced. Applying extrapolation approaches similar to those used in bulk sequencing studies allows us to derive richness estimates from the relative rates of occurrence of those rare doublets compared to the majority of singletons. We applied the Chao1 estimator [23], which is a non‐parametric measure frequently used to estimate empirical population richness from a limited sample, to the naïve T cell receptor repertoire sequences from the TABLO data. Estimates from each donor yield richness estimates ranging from ~10^8^ clonotypes in children to ~10^7^ in adults (Figure 3B). These values fall at the upper range of previous estimates but agree with those derived by the more robust experimental approach of replicate bulk sequencing [21]. Interestingly, both the earlier and the recent study reported an age‐dependent drop in repertoire richness.

An important, though sometimes overlooked, fact is that extrapolated richness estimates should be interpreted as lower bounds on true richness [24, 25], particularly when clone frequencies are heterogeneous. To illustrate this, we apply the Chao1 estimator to two hypothetical scenarios: First, a naïve repertoire comprising 10^8^ clones, each consisting of 1000 cells; and second, a naïve repertoire containing a mixture of 10^4^ clones of 100,000 cells each, and 9.9 × 10^10^ singleton clones. In both cases, we simulate sampling 100,000 cells from a total repertoire of 10^11^ cells and apply the Chao1 richness estimator to the simulated data. Results from 100 repetitions of this procedure are shown as histograms in Figure 3C,D. In the even distribution, the estimated richness closely recapitulates the true richness of the underlying repertoire (Figure 3C). In the uneven community, however, the estimated richness was equivalent to that of the even community (Figure 3D), despite the true richness being three orders of magnitude larger. As we discuss in detail below, there is good evidence for substantial heterogeneity in clonal frequencies in the naïve repertoire, and true richness is thus likely to considerably exceed these lower bounds.

On the basis of all the above, it is reasonable to conclude that the size or richness of the naïve repertoire of adult humans exceeds 10^8^ distinct molecular TCR clonotypes. Assuming a naïve compartment of 10^11^ T cells [26], this gives an average clonotype family size of 1000 cells.

### 1.2 Generation of Diversity

What limits are imposed on the size of the naïve repertoire by the mechanism of generation of diversity? We first consider the theoretical maximum potential size of the TCR alpha/beta repertoire. The heterogeneity in T cell sequence/structure arises from imprecise somatic recombination of multiple V, J, and (for beta chains) D regions. The number of functional V and J regions varies between individuals (see https://www.imgt.org/IMGTrepertoire/LocusGenes/genetable/human/geneNumber.html#functional). It is also difficult to accurately determine the maximum number of deletions or additions that occur during junction formation, since the probabilistic process of recombination can result in the same sequence via different mechanistic series of steps. However, Murugan et al. [27] have estimated the probabilities of each step of T cell receptor recombination using a maximum likelihood framework. Using their values for the maximum number of deletions and additions which are observed in experimental data, and the maximum number of V and J genes reported, allows an approximation of the maximum possible repertoire size of 7.7 × 10^46^ possible distinct DNA sequences (Table 1). The redundancy of the genetic code and the requirement that the recombination gives rise to a productive protein sequence will reduce the number of protein sequences by orders of magnitude [28]. However, this number of TCRs remains large with respect to the total number of T cells in the immune system, which is estimated to be in the order of 4‐5 × 10^11^ [29, 30, 31].

**TABLE 1:** The number of variable genes, non‐template deletions, and additions observed in the human TCR repertoire (mostly from Murugan et al. [27]). The Table also shows the calculated number of possible combinations of these processes, with and without non‐template additions.

Chain	Segment	# Genes	Max deletions	Max insertions	Combinations	Total combinations
Alpha	V	45	12	—	3.24 × 10^5^	3.5 × 10^17^
	J	50	12			
	VJ	—	—	20	1.1 × 10^12^	
Beta	V	48	12	—	1.8 × 10^5^	2.2 × 10^29^
	D	2	—			
	J	13	12			
	VD	—	—	20	1.1 × 10^12^	
	DJ	—	—	20	1.1 × 10^12^	

**FIGURE 4:**
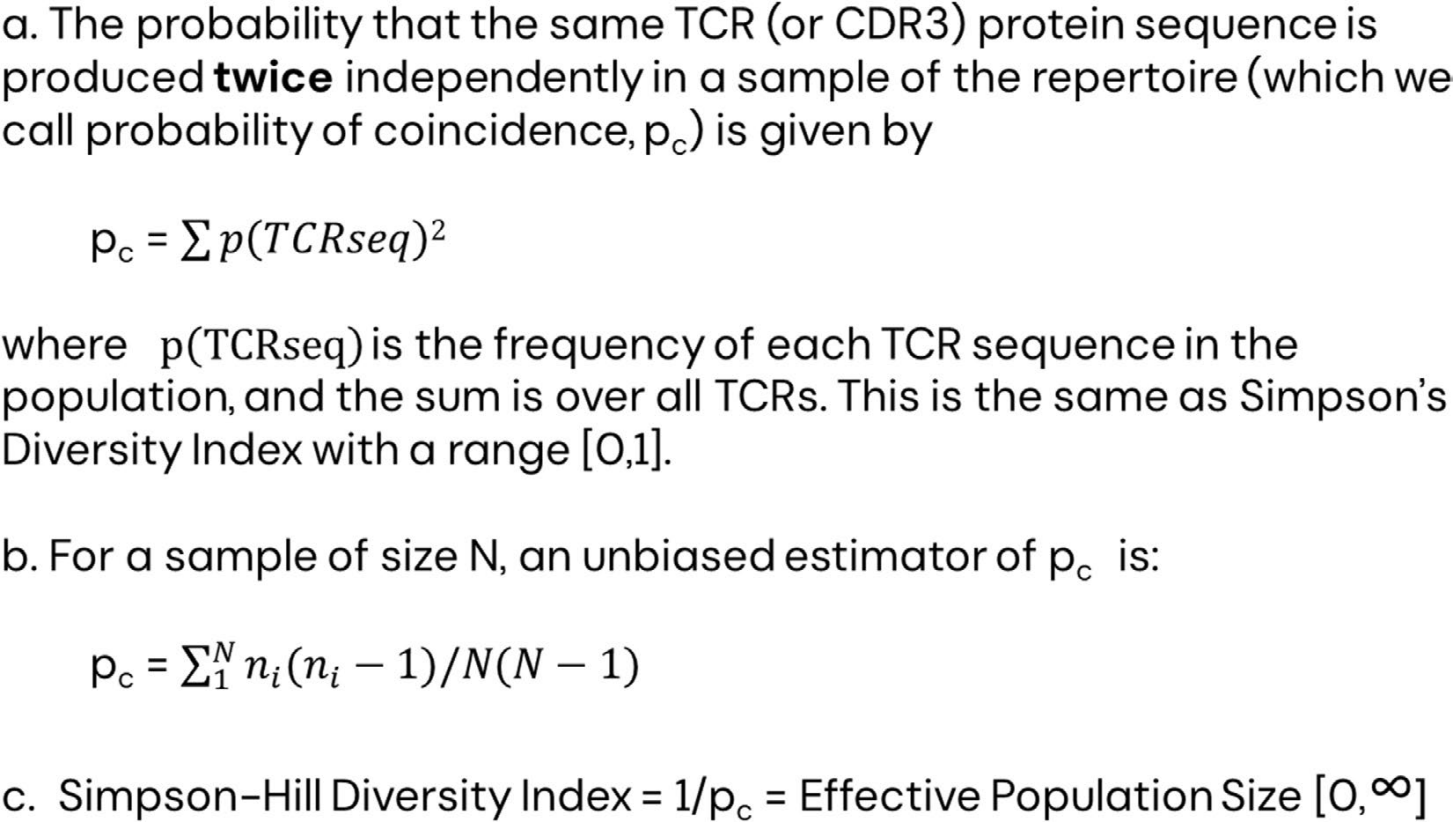
The key equations defining the ‘probability of co‐incidence’, and how these relate to the Simpson's Diversity index and the effective population size. (A) Population‐based definition of probability of coincidence as a measure of clonal convergence. (B) Estimation of this quantity from a finite sample using an unbiased estimator. Here, n_i is the number of observed distinct nucleotide sequences coding for TCRseq i, and *N* = sum n_i is the total number of observed clones. (C) The inverse of the probability of coincidence is a diversity measure for the effective number of TCR sequences in the repertoire.

This simplistic calculation for the maximum number of TCR sequences which can be generated by the somatic recombination mechanism illustrated in Table 1 ignores the fact that the probability that each clonotype is produced varies over a huge range, since the usage of different V or J genes, the number of deletions, and the number of additions are all non‐uniform [27]. A more sophisticated approach to estimating the maximum potential size of the repertoire is to consider the ‘effective’ population size, which estimates the population size at which TCRs will begin to be ‘resampled’, and hence population diversity will begin to be limited. A theoretical framework for this analysis was developed by Mayer and Callan [32], who introduced the ‘probability of coincidence, Pc’, the probability of observing TCR clonotypes twice in a repertoire (Figure 4). Intuitively, the inverse of this probability reflects the effective number of species in the repertoire. When a sample is small enough that every species is only represented once (Pc = 0), one can say nothing about the total number of species, which effectively appears infinite to the observer. When a sample of a population is large enough that every species is observed at least twice, the sample has effectively captured all the possible species in the population. This measure has, in fact, been studied extensively as a measure of diversity in ecology (Simpson's Diversity Index) [33]. The inverse of Simpson's diversity index (sometimes referred to as the Simpson‐Hill diversity) is similar to the concept of effective population size commonly used in evolutionary genetics [34, 35].

**FIGURE 5:**
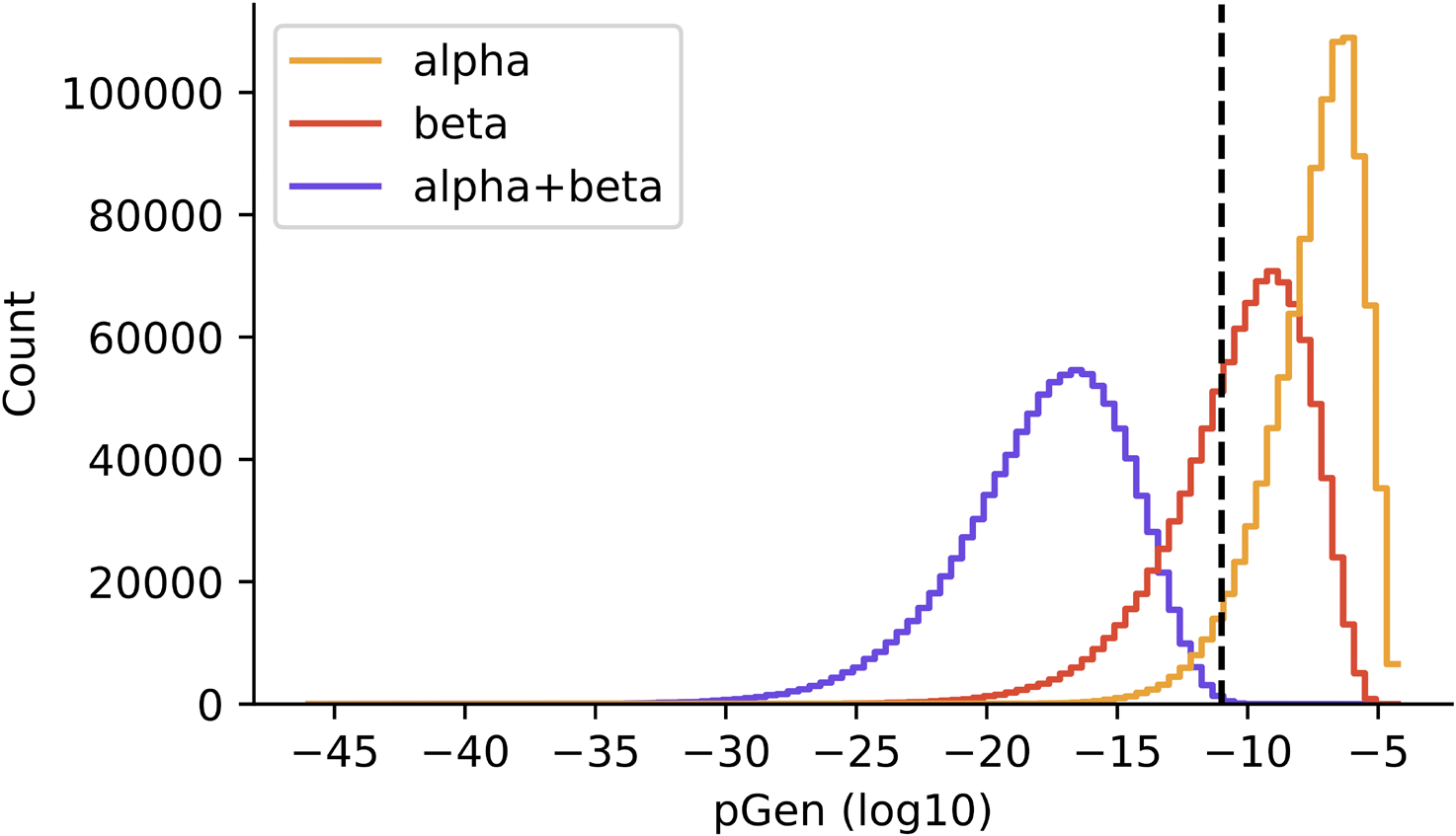
The probability of generation of 10^6^ synthetic TCRs, created without external selection. 10^6^ individual TCRs were created using the OLGA algorithm [28], according to recombination probabilities calculated from experimental data in the absence of antigen selection [27]. The plot shows a frequency histogram of probabilities for TCR alpha and TCR beta, and TCR alpha/beta heterodimers, calculated by selecting a random alpha and beta chain, and multiplying their generation probabilities. The vertical dotted line shows the estimated number of cells that make up the human naïve T cell compartment.

How can we estimate the potential Simpson's Diversity and effective population size of the TCR repertoire, taking into account the non‐uniform probabilistic nature of somatic recombination? The probabilistic model for TCR somatic recombination developed [27] for nucleotide sequences has been extended to protein sequences using dynamic programming to efficiently sum over possible recombination scenarios that give rise to the same clonotype [28]. Because the underlying model of recombination was learnt using non‐productive out‐of‐frame recombination events, which are created during the recombination process but are not expressed as proteins and therefore are not subject to biological selection, this allows an estimate of repertoire generation parameters unaffected by selection. The algorithm, named OLGA, can be used as a generative model that creates artificial repertoires according to the pre‐learned parameters fitted to the experimentally observed recombination outcomes. The probability distributions of an artificial repertoire of 10^6^ alpha and beta chains created independently using OLGA are shown in Figure 5. Since there is little evidence of alpha‐beta pairing constraints in naïve repertoires [36], the probability of individual clonotypes can be calculated simply by multiplying the probabilities of the two chains. The overwhelming majority of the distribution for the combined alpha/beta clonotypes lies at probabilities < 10^−11^. Given that the number of naïve T cells is in the order of 1‐2 × 10^11^ [26], identical clonotypes will therefore be recombined only very rarely. One can calculate the effective population size of this theoretical ‘unconstrained’ repertoire by calculating the Simpson's diversity of this artificially generated repertoire. Conveniently, an unbiased sample estimate of Simpson's diversity [33] and of its variance [37] exists (Figure 4B). Using this estimator, we arrive at an estimate of Pc = 4.8 × 10^−14^, giving an effective potential TCR repertoire population size of 1/Pc = 2 × 10^13^. This number is much smaller than the total number of species shown in Table 1. However, it is still much larger than the number of T cells in the naïve repertoire. The richness of the observed repertoire is therefore not limited by the mechanism of somatic recombination, but by other subsequent processes such as convergent thymic selection and clonal expansion.

It is instructive to compare this measure of effective repertoire size for an artificially created and therefore unselected repertoire with the same measure calculated on an actual observed TCR repertoire. Two large‐scale sets of single‐cell peripheral blood naïve cells have been published [18, 38]. The probability of coincidence for naïve T cells in HLA‐matched individuals in the first set has been calculated previously as 2 × 10^−9^ [32], translating into an effective repertoire size of 5 × 10^8^. The probability of coincidence for the second set (the same data for which we calculated richness above) can be calculated as 1.9 × 10^−9^. This translates into an effective repertoire estimate of 5.4 × 10^8^, similar to the previous estimate. Both estimates of effective repertoire size are substantially smaller than the effective size of the theoretical repertoire constructed simply from the probabilistic model of somatic recombination. Thymic selection may be the major contributor to driving this repertoire convergence.

**FIGURE 6:**
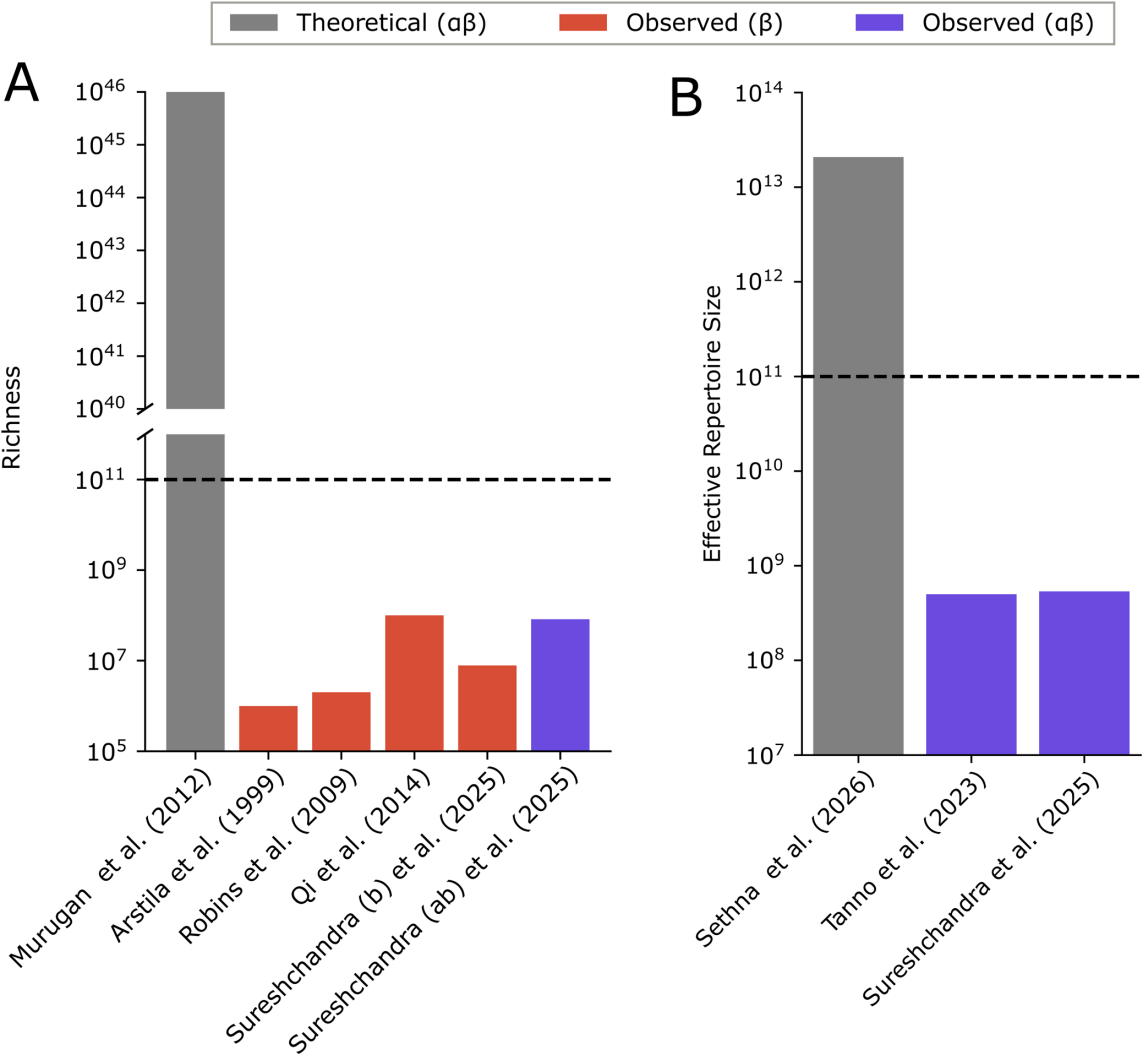
A summary of estimates of repertoire size. (A) Repertoire richness, taken from [18, 20, 21, 22]. (B) Effective repertoire population size, taken from [18, 38], and the estimate of effective repertoire size in PBMC using the probability of coincidence framework and large‐scale single‐cell sequencing [32].

We summarize the estimates of repertoire richness and effective repertoire population size in Figure 6. Both richness and effective repertoire size are greater than 10^8^, but are much smaller than the theoretical maximum.

### 1.3 The Frequency Profile of the naïve Immune Repertoire: Are All naïve Clonotypes Equal?

In the previous section, we concluded that the richness of the naïve repertoire is not limited by the somatic recombination mechanisms that, even taking into account the biases of the system, rarely create identical molecular clonotypes in an individual.

However, the number of cells carrying a specific TCR (which we will refer to as the clonotype family size) is determined by several processes subsequent to the recombination events themselves. Thymocytes may divide in the thymus post‐selection, giving rise to identical clonotypes of variable sizes, or after leaving the thymus and entering the naïve repertoire. Thymocytes and naïve cells may also undergo cell death, thus reducing the number of members of a particular clonotype. Most proliferation in the thymus occurs before selection, and before definitive expression of a single alpha/beta TCR on the cell surface. However, some studies [39] have suggested that single positive fully selected thymocytes undergo a ‘few rounds’ of proliferation before exiting the thymus into the periphery, or immediately after exiting [40]. There seems to be a paucity of more recent data either confirming or contradicting this observation.

Proliferation in the naïve compartment of T cells in the periphery has been studied in detail both in mice and humans (reviewed in [41]). Because of the longer lifetimes of naïve human T cells, the thymus continues to play an important role in replenishing the T cell naïve repertoire in mice more than in adult humans [42]. In principle, knowledge of naïve T cell turnover kinetics would allow estimation of the predicted frequency distribution of the naïve repertoire. However, measurements of T cell kinetics are usually inferred from metabolic labeling of dividing cells, giving only average population kinetics, and very little information on the variance of proliferation and death rates between clonotypes. It is this variance, however, which is the key potential determinant of the heterogeneity of the clonotype family size of the naïve repertoire [43]. The existence of heterogeneity in turnover kinetics has been proposed in mice [44], with the authors describing the presence of a long‐lived population of naïve cells established early in life.

A detailed analysis of the heterogeneity of clonotype family size in naïve repertoires is presented in [45]. The study uses single‐chain TCR sequence data generated from naïve T cell populations enriched by fluorescent cell sorting of human peripheral blood T cells. Since sequencing is performed on populations of T cells (bulk sequencing) rather than individual cells (single‐cell), the pairing between alpha and beta sequences is lost, and the analysis of each chain has to be carried out separately. The study examines and attempts to mitigate several possible confounders, such as the ‘naïve’ population purity and the presence of multiple RNA molecules from each T cell. The study finds evidence that some TCR sequences are present at high frequency (10^−6^—10^−5^), suggesting clones of ‘naïve’ cells as large as 10^5^–10^6^ (assuming a naïve compartment of 10^11^ cells). Despite substantial sampling effort, the largest clonotypes were still only present a few times, and all other clonotypes were seen only once. While even such modest copy numbers within a small sample strongly support the notion of a broad range of naïve clonotype sizes, the data were not sufficient to accurately specify the full distribution. However, mathematical modeling suggests that simple birth/death models of clonal proliferation using known average naïve T cell turnover rates do not predict the spread of clonotype sizes necessary to explain the observations. A subsequent, more detailed modeling [43] proposed a refinement of the birth/death model in which proliferation rates varied with clonotypes. In this setting, even a modest heterogeneity in proliferation rates leads to wide divergence in predicted clonotype family size, consistent with an underlying power law distribution [46]. The mechanisms driving power law distributions in T cell receptor repertoires have been investigated [11, 13, 46, 47], but most studies focus on memory/effector populations where much higher clonal frequencies allow more accurate inference of frequency distribution parameters.

As discussed in the section on diversity, studies agree that the functional clonotype size of the populations of T cells recognizing a specific antigen can vary by several orders of magnitude, depending on the peptide/MHC construct [12]. However, the actual estimates of the population sizes vary widely, perhaps reflecting differences in methodology and specificity. There is also disagreement on whether differences in precursor numbers determine the magnitude of the subsequent immune response [5]. Heterogeneity in functional clonotype size can arise from differences in the number of T cell clonotypes within each functional set, or the number of cells carrying each molecular clonotype identified. Although there is general agreement that the repertoire of T cells binding to a single epitope is diverse, with as many as 500 different TCR sequences for a single epitope [12, 48], these studies do not directly address the molecular clonotype family size distribution.

**FIGURE 7:**
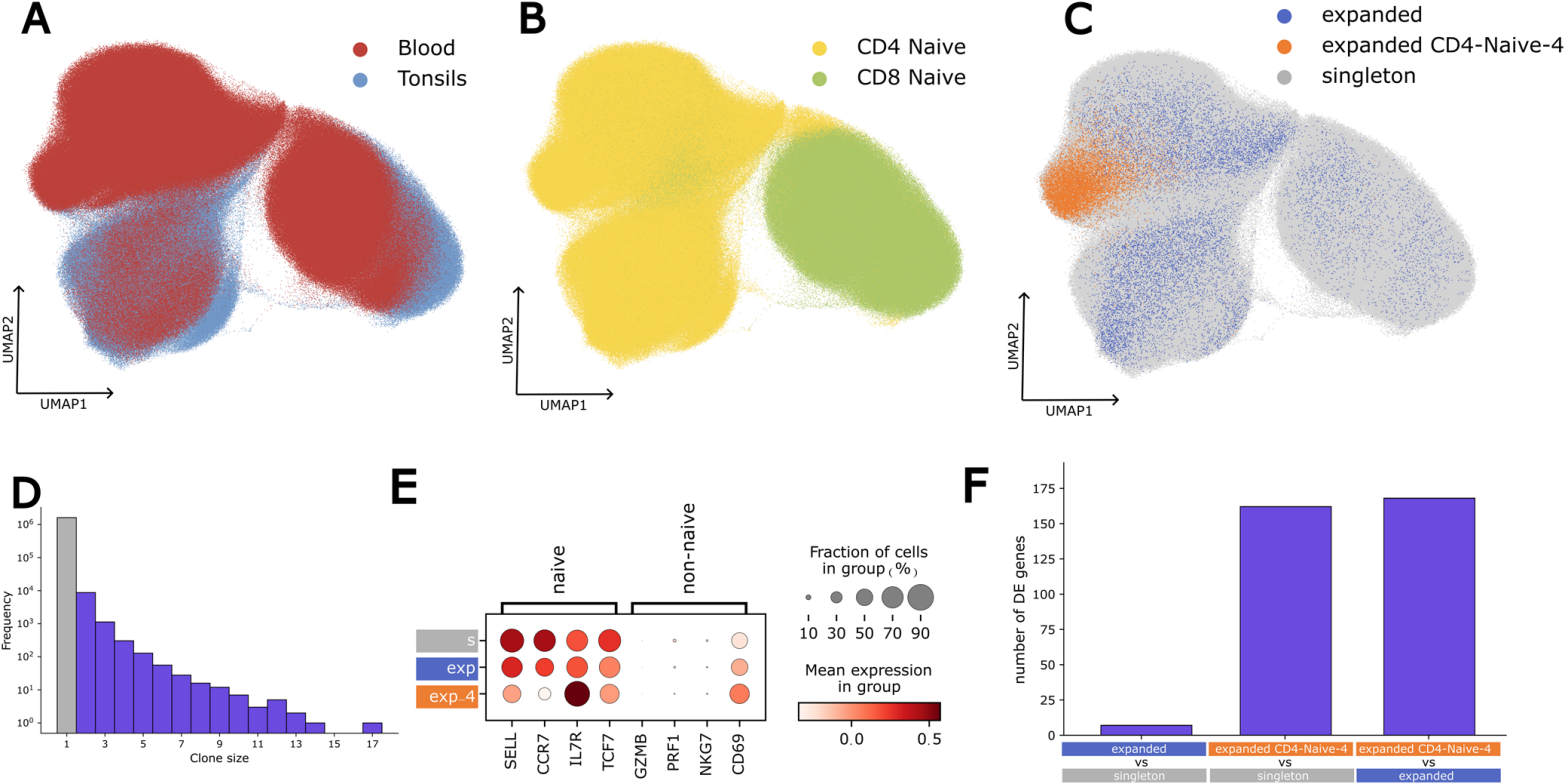
The TABLO sc‐RNA sequencing atlas contains 5.7 million T‐cells from human blood and tonsil tissue from 10 donors [18]. 1.6 million of these cells were defined as naïve T‐cells in the original study’s annotation. (A–C) UMAP of naïve T‐cells split by tissue compartment, CD4 and CD8 phenotypes, and TCR clone expansion status. A cell is a singleton if no other cells were found in that donor with the same TCR nucleotide sequence. A cell is expanded if more than one cell has the same TCR nucleotide sequence. The CD4‐Naïve‐4 cluster was enriched for expanded clones and thus analyzed as a separate compartment. (D) Histogram of clone sizes of all cells annotated as naïve. (E) Gene expression of naïve and non‐naïve markers for T‐cells. (F) The number of differentially expressed genes between singletons, expanded, and expanded CD4‐naïve‐4 cells. Cells were pseudo bulked by calculating the mean log‐normalized expression per donor and performing a paired *t*‐test across donors. Singletons were subsampled to have a similar number of cells. A gene was considered differentially expressed if the change in average expression was greater than 0.25 and the *p*‐value < 0.05 (Benjamini Hochberg correction).

The deeply sequenced single‐cell TABLO dataset [18], from which we derived richness estimates in a previous section, can also be used to identify clonal expansions within the naïve repertoire. The naïve compartment was made up of cells found in both the blood and tonsil and CD4 and CD8 phenotypes (Figure 7A,B). The clonal frequency of all cells annotated as ‘naïve’ in the published analysis is shown in Figure 7D. Although the majority of clonotypes were seen only once, consistent with a frequency of 1 in a million or less, some ‘naïve’ TCRs were seen two or more times. Approximately half of these ‘high frequency’ cells were part of the CD4‐naïve‐4 cluster and form a specialized sub‐compartment of cells all originally classified as ‘naïve’ (Figure 7D). The expanded CD4‐naïve‐4 cells still express some naïve markers, such as L‐selectin (SELL), but do not express CCR7 at high levels and have higher expression of the activation marker CD69 (Figure 7E). Overall, their phenotype is quite distinct from the remainder of the naïve population, with over 150 differentially expressed genes compared to the singletons and other expanded naïve cells (Figure 7F). It is difficult to determine whether this is a sub‐population of antigen‐experienced cells mistakenly co‐clustered with the genuine naïve repertoire or whether these cells represent a unique subpopulation of genuinely ‘naïve’ cells that have not been exposed to cognate foreign antigen, but which have undergone more extensive proliferation. Interestingly, the remaining ‘high frequency’ cells are distributed remarkably evenly across the embedding space, have similar expression of naïve markers compared to singletons, and have less than 10 DEGs with singletons (Figure 7C–F). Some of these clones are sampled more than twice, suggesting clonal frequencies greater than 1 in 10^6^ , corresponding to clone family sizes of greater than 10^5^ cells. Taken together, the data exemplifies the difficulty in defining a naïve cell. However, it also supports the hypothesis that the naïve repertoire spans a broad range of T cell clonotype family sizes.

Two recent studies proposed the existence of large clonotype families in the naïve (or pre‐exposure) immune repertoire that respond to infectious disease challenges. The first [49] analyzed the repertoire of responding T cells in a longitudinal study of health workers during the early stages of the SARS‐CoV‐2 pandemic. The study found that a number of T cell clonotypes expanded rapidly very early following infection. These clonotypes were found to be present at high frequency in pre‐pandemic repertoires, although it was not possible to unambiguously distinguish between the presence of large naïve clonotypes or high frequency memory cells cross‐reactive for some other antigen (e.g., circulating human coronaviruses) in this study. The same paper also reported the presence of high‐frequency LCMV‐specific T cells in mice pre‐exposure, suggesting that the existence of large precursor clonotypes might be a general feature of naïve T cell repertoires.

A second, more recent study [50] analyzed the changes in T cell clonotypes in a human challenge study, in which healthy young adults, seronegative and presumed unexposed to SARS‐CoV‐2, were infected with the SARS‐CoV‐2 virus (Wuhan strain) in a controlled human challenge setting. Blood samples were collected daily, and TCR repertoires sequenced. As in Milighetti et al., TCR clonotypes emerged rapidly within a few days of infection. Mathematical modeling suggested the heterogeneity in clonotype trajectory observed could again be parsimoniously explained by variation in naïve precursor clonotype family size.

In summary, the clonotype frequency distribution in the naïve cell repertoire remains very poorly defined. Major experimental challenges are the robust classification and identification of naïve vs. antigen‐exposed cells. The absence of prior exposure to a specific antigenic epitope can be confounded by the potential for T cell cross‐reactivity [6, 7]. In humans, these challenges are compounded by the fact that typical sample sizes of a few million T cells represent less than 1 in 10^5^ of the total number of T cells in an individual. Nevertheless, the balance of available evidence suggests that naïve clonotype family size is heterogeneous in the human repertoire, with potential upper bounds in the order of 1 in 10^5^. The bulk of naïve T cells are present at less than 1 in 10^6^; however, even this would correspond to a family clonotype size of 100,000 T cells. The shape of the lower part of the distribution, constituting over 90% of the naïve repertoire, remains completely unknown. In the mouse, which is somewhat more tractable to direct experimental investigation, clonotype family sizes are much smaller. However, variation between 1 and a few hundred T cells per clonotype may exist.

### 1.4 The Inclusivity of the naïve T Cell Repertoire

Just as clonal expansion lies at the core of the theory of adaptive immunity, so clonal deletion has long been the cornerstone of adaptive immune tolerance. The key idea is that cells exposed to self‐antigen at some specific step in their development/differentiation pathway enter apoptosis, and hence ‘holes’ appear in the mature repertoire such that cells specific for each self‐antigen are absent from the naïve repertoire. This mechanism leads to the absence of self‐reactivity, or self‐tolerance (Figure 1B). The theory was originally developed in the context of B cells and antibody production [1]. However, it rapidly became apparent that B cells specific for many self‐antigens were present, but were silent in the absence of linked relevant T cell help [51, 52, 53]. Although examples of B cell clonotype deletion exist, especially to high concentration autoantigens like albumin [54], the focus for clonal deletion turned to T cells, and especially to the role of negative selection in the thymus. The early evidence showing direct evidence of clonal deletion of T cells during thymic selection has been summarized in Goodnow [55]. The first set of experiments rests on T cell transgenic mice, in which a single TCR is expressed in all T cells of a mouse. Under these conditions, if the TCR introduced is specific to a self antigen, extensive T cell death of double‐positive thymocytes occurs, and very few mature T cells exit into the periphery [56, 57, 58]. This approach has been extended to the human TCR using a transgenic humanized mouse model [59]. A second set of experiments used superantigens, an unusual class of antigens that activate T cells not via classical peptide/MHC binding with the CDR loops of the receptor, but via direct binding to the V beta proteins. The key examples of such superantigens in mice were later shown to be derived from endogenous retroviruses and mouse mammary tumor viruses, carried in specific strains of laboratory mice [60]. Investigation of thymic development in mice carrying these endogenous superantigens showed that T cells carrying the cognate V beta genes were deleted during the double‐positive thymic developmental stage (reviewed in Simpson et al. [61]). Both these lines of evidence proved very persuasive, and the idea that clonal deletion is fundamental to the mechanisms of central thymic tolerance is standard dogma in most immunology textbooks today. However, these experimental systems differ in a number of significant ways from the single peptide/MHC complex‐induced tolerance, which determines the majority of the repertoire. Transgenic T cells are present at far higher precursor frequencies than normal T cells, and superantigens interact in a non‐canonical way with the TCR to induce intracellular signaling.

The predominance of clonal deletion as the major mechanism driving central tolerance persisted for more than 10 years since these pioneering experiments. Nevertheless, a number of studies eventually started to question the dogma of thymic deletion as the major mechanism of T cell self‐tolerance [62] studying mouse CD4 cells, and [63] studying human CD8 cells, failed to find convincing evidence of functional ‘holes’ in the repertoire for self‐antigens. The distinction between clonal deletion at a functional and individual clonotype level has been highlighted by [64]. In a study focused on a single self‐antigen recognized by CD4+ mouse T cells, the predominant mechanism of self‐tolerance was differentiation of self‐reactive thymic Treg cells, but the study did not rule out deletion of some individual clonotypes.

It is worth considering the technical challenges in observing ‘holes’ in the repertoire, which have made reliable progress in this area so difficult. The basic problem lies in the huge diversity and hence low frequency of individual TCR clonotypes, or even functional antigen‐specific clonotypes in the naïve repertoire. Functional T cell assays, such as proliferation or cytokine secretion, can have enormous sensitivity since they typically involve exponential T cell growth, and have been used as surrogates of T cell presence/absence [65]. However, since T cells can exist in anergic states or be silenced by Treg activity, direct observation is needed to unambiguously document clonotype deletion. The direct detection of antigen‐specific T cells within the naïve repertoire, by binding to soluble pMHC multimers, required complex and lengthy enrichment protocols [66]. In fact, individual mice were found to contain only around a few hundred T cells specific for most epitopes, although the frequency of antigen‐specific cells varied by two orders of magnitude. At the molecular clonotype level, the technical limitations are even greater. Since the diversity of the repertoire is such that most alpha/beta pairs are likely to be unique to that individual (private), the absence of specific clonotypes is usually due to stochastic recombination rather than deletion. Comparing pre‐selection to post‐thymic selection in the same individual, which can be done in the mouse, can partially mitigate this problem. The introduction of fixed TCR alpha, or fixed TCR beta chains in transgenic mice has been used to reduce the overall diversity of the TCR repertoire, e.g, [64]. albeit at the price of introducing some artificiality. None of these mitigation approaches can be used in humans. In conclusion, direct experimental evidence that thymic selection causes true functional ‘holes’ in the repertoire by deletion of all TCRs against specific peptide/MHC complexes remains quite limited. Functionally, however, the activity of T cells against self‐targets is limited by a variety of mechanisms, including anergy and Treg activity.

A different approach to the study of negative selection has relied on population‐based measurements, often coupled with mathematical modeling, to infer excess cell death during thymic selection. A core objective has been to partition the drivers of this excess cell death between negative selection by deletion and the absence of positive selection (death by neglect). Much of the literature on this topic has been reviewed in detail by [67, 68]. Interestingly, a much more recent review [69] relies heavily on older literature in discussing clonal deletion. The overall conclusion emerging from these studies [70, 71] is that, at least in the mouse, a specific sub‐population of dendritic cells within the thymic medulla induces thymocyte cell death. However, linking this death to the deletion of specific clonotypes specific for individual self‐antigens to create ‘holes’ in the repertoire has remained challenging and requires additional research.

The existence of negative selection has also been studied at the level of the TCR repertoire [72]. The study combines TCR sequencing (unpaired alpha and beta gene bulk sequencing) with multiparameter flow cytometry to identify, separate, and TCR sequence key thymocyte subpopulations. The transcription factor *nur* was used to specifically isolate thymocytes undergoing selection via TCR/pMHC binding. Differences were clearly detected between TCR repertoires at different stages of selection, reflecting a gradual decrease in diversity during differentiation toward single positive mature T cells. Positive selection was also detected, since CD4 MHC class II TCRs could be distinguished at a population level from CD8 MHC class I TCRs, although there was substantial overlap in individual single‐chain sequences within both populations. However, more detailed analysis searching for specific motifs or CDR3s that were absent from mature populations, suggestive of antigen‐specific ‘holes’ in the repertoire, could not be detected. The study concludes that negative selection of single positives is only weakly associated with the sequence properties of single TCRs, or at least single chains. It is, however, possible that larger differences exist in the paired alpha‐beta repertoires, which would not be detectable in either the alpha or beta repertoires alone. While previous work on the functional alpha‐beta repertoire has suggested that pairing in the naïve repertoire was largely random, with weak associations between germline genes [73, 74], there is now substantial quantitative evidence for strong pairing constraints among TCRs specific to foreign antigens [36]. Such constraints also likely apply to the interactions of TCR with negatively selecting self‐pMHC, which might mask holes when considering the distributions only at the single‐chain level.

We conclude that there is rather little evidence that individual self‐pMHC complexes induce efficient clonal deletion of all self‐reactive T cells in the thymus, and hence induce ‘holes’ in the repertoire. Even from a theoretical perspective, given the timescales of thymic selection [67], the speed and random walk of medullary thymocytes [75] and the stochastic nature of self‐antigen expression in individual medullary cells, it has been suggested that it is unlikely that a given TCR will be exposed to all possible AIRE‐dependent and AIRE‐independent self‐peptides presented by epithelial or dendritic antigen‐presenting cells in the thymus. Rather, if each cell probes a random subset of all expressed self‐antigens, and the numbers of presented peptides vary for different cells, each TCR may probe only 1%–5% of self‐peptides in humans. Thus, individual thymocytes could remain cross‐reactive to a majority of self‐peptides, severely limiting the role of negative selection in maintaining self‐tolerance. It also implies that each TCR experiences its own private selection linked to the particular set of self‐peptides it encountered. Nevertheless, the definition of ‘self’ at a functional clonotype level must be very precise since many pathogen epitopes are only one amino acid away from their closest ‘self’ peptide [76]. This argument suggests we should not expect to see strong signatures of eliminated TCRs due to negative thymic selection in the repertoire, even at the level of full alpha/beta molecular clonotypes.

**FIGURE 8:**
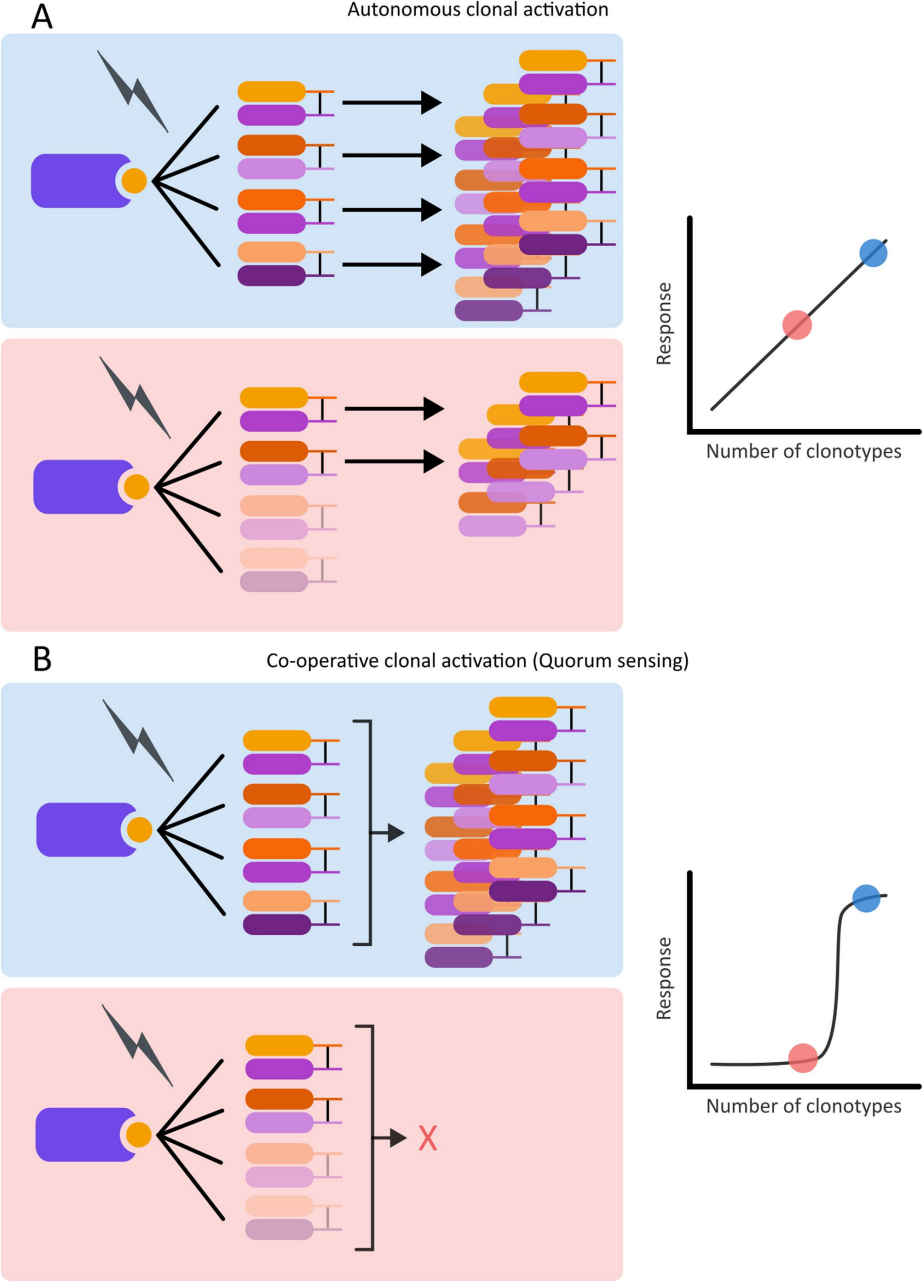
Schematic representation illustrating the impact of partial clonal deletion on the overall immune response. (A) If T cells respond to an antigen autonomously, partial deletion (lower panel) leads to a quantitative decrease in response. (B) If T cells require clonal co‐operativity to respond to an antigen (quorum sensing), partial clonal deletion can give rise to a complete absence of response.

Both experimental and theoretical considerations therefore suggest that T cell clonal deletion is at best not complete, yet self‐tolerance is usually robust and persistent. Two general forms of solution have been suggested to address this apparent paradox. The first is that recognition of self‐pMHC drives differentiation of Tregs, high‐affinity T cells specific for specific self‐antigens, which can provide dominant suppression of any self‐specific T cells that escape selection and develop into naïve T cells. The field of thymic Tregs lies outside the scope of this review, but has been abundantly reviewed elsewhere (e.g., [77]). A second different solution was explored in [78], who propose that self‐tolerance exists not at the level of individual T cells, but rather at the level of a T cell ‘quorum’ (Figure 8). In this model, sensing and reacting to an antigen, whether self or non‐self, requires the concerted action of a population or cohort of T cells [79], [78] propose that quorum sensing can generate self‐tolerance which is highly resilient even in the presence of significant escape from clonal deletion. The two mechanisms are, of course, not mutually exclusive, and further research will be required to quantitatively determine the importance of one, the other, or both mechanisms in specific examples of self‐tolerance.

Before concluding this discussion of repertoire ‘inclusiveness,’ we would like to briefly review another situation in which ‘holes’ in the repertoire have frequently been invoked to explain well‐documented experimental observations. The decreasing effectiveness of the immunity in old age has been extensively documented (see the set of articles in [80, 81, 82]). A number of studies (reviewed in [41]) have also shown that the diversity of the immune repertoire falls with age, as more cells transit from naïve to memory and effector phenotype, and the repertoire ‘fills up’ with highly expanded antigen‐experienced cells. The extent and age at which the diversity of the naïve repertoire falls is still a matter of debate, with some studies suggesting a gradual decrease with increasing age [83], while others suggest a more dramatic fall in diversity above 70 [21]. The CD8 T cell naïve compartment may be more affected than the CD4 cells. Interestingly, a recent study of the repertoire of wild mice found no clear relationship between diversity and age [84]. This is somewhat counterintuitive since the thymus contributes much more to maintaining the repertoire in mice [42]. Given the parallel fall in T cell immunity [85] and the fall in T cell diversity, it is tempting to propose that T cell immunity in old age is indeed limited by the limited diversity of the naïve repertoire. The failure to respond, according to this hypothesis, is due to the gradual emergence of ‘holes’ in the repertoire against novel antigens [86, 87]. However, as discussed in the first part of this review, and pointed out in [41, 81], the human repertoire is enormous, and many individual clonotypes are likely to contribute to the overall immune response to any epitope. Furthermore, several mechanisms have been described that can protect against age‐related repertoire erosion [88, 89]. It therefore remains difficult to measure the extent to which naïve repertoire erosion contributes to immunosenescence. In contrast, multiple alternative mechanisms, including changes in migration [90] and cellular metabolism [91] may all contribute to non‐responsiveness in the elderly.

## 2 Conclusion

The last 10–15 years have seen intense study of the T cell repertoire, driven in large part by the capabilities of massively parallel DNA sequencing, and more recently by the advent of accurate single‐cell sequencing. Despite all this work, several key questions remain unsolved. In the first part of our review, we discuss the ongoing debate on the richness of the population of T cell clonotypes that exist in an individual repertoire. Although the bias of the recombination machinery somewhat restricts repertoire **Diversity**, the independent recombination of TCR genes to give identical protein sequences in one repertoire is very rare and does not contribute significantly to clonotype inequality. The richness of the human TCR repertoire is greater than 10^8^, leading to largely idiotypic but robust responses to most foreign antigens in most healthy individuals. The functional properties of the repertoire are determined not only by richness but by the frequency distribution of clonotype family size (**Equality**). The majority of clonotypes are present at frequencies of less than 1 in 10^6^, and there is therefore little experimental evidence regarding the actual size of these clones, which could theoretically range from 1 to 10^5^. However, both theoretical and experimental evidence point to the existence of a broad range of family clonotype sizes, with a small number of naïve TCRs present in more than 10^5^ cells. The mechanisms that drive naïve clone size heterogeneity remain almost entirely speculative. The impact of naïve clonotype size inequality on the functional dynamics of primary immune responses also requires further study. Finally, we discuss the contribution of clonal deletion to self‐tolerance and age‐related decrease in immunocompetence (**Inclusion**). We conclude that there is rather limited evidence that a failure to respond to an epitope is due to deletion of the family of clonotypes that potentially respond to that epitope (a ‘hole’ in the repertoire), and therefore, clonal deletion may have a limited role in both processes.

## Funding

This work was supported by Cancer Research UK, Rosetrees Trust, and Wellcome Trust.

## Conflicts of Interest

The authors declare no conflicts of interest.

## Data Availability

The data that support the findings of this study are available in Zenodo at https://doi.org/10.5281/zenodo.13119615 (TCR info) and https://doi.org/10.5281/zenodo.17308874 (GEX data).
